# Obesity Associated Cerebral Gray and White Matter Alterations Are Interrelated in the Female Brain

**DOI:** 10.1371/journal.pone.0114206

**Published:** 2014-12-10

**Authors:** Karsten Mueller, Annette Horstmann, Harald E. Möller, Alfred Anwander, Jöran Lepsien, Matthias L. Schroeter, Arno Villringer, Burkhard Pleger

**Affiliations:** 1 Max Planck Institute for Human Cognitive and Brain Sciences, Leipzig, Germany; 2 Integrated Research and Treatment Center (IFB) Adiposity Diseases, Leipzig, Germany; 3 Clinic of Cognitive Neurology, University of Leipzig, Leipzig, Germany; Wake Forest School of Medicine, United States of America

## Abstract

Obesity is known to affect the brain's gray matter (GM) and white matter (WM) structure but the interrelationship of such changes remains unclear. Here we used T1-weighted magnetic resonance imaging (MRI) in combination with voxel-based morphometry (VBM) and diffusion-tensor imaging (DTI) with tract-based spatial statistics (TBSS) to assess the relationship between obesity-associated alterations of gray matter density (GMD) and anisotropic water diffusion in WM, respectively. In a small cohort of lean to obese women, we confirmed previous reports of obesity-associated alterations of GMD in brain regions involved in executive control (i.e., dorsolateral prefrontal cortex, DLPFC) and habit learning (i.e., dorsal striatum). Gray matter density alterations of the DLPFC were negatively correlated with radial diffusivity in the entire corpus callosum. Within the genu of the corpus callosum we found a positive correlation with axial diffusivity. In posterior region and inferior areas of the body of the corpus callosum, axial diffusivity correlated negatively with altered GMD in the dorsal striatum. These findings suggest that, in women, obesity-related alterations of GMD in brain regions involved in executive control and habit learning might relate to alterations of associated WM fiber bundles within the corpus callosum.

## Introduction

Obesity is associated with structural alterations of regions in the frontal cortex and limbic system [1], increased prevalence of white matter (WM) lesions, and declined memory and executive functions [2], [3]. Comparable deteriorations occur with advancing age suggesting that obesity accelerates brain aging [4], [5].

Besides these rather widespread neurodegenerative-like processes, obesity specifically affects function and structure of brain regions involved in reward processing. In obese individuals, high-caloric food seems to amplify hedonic responses from key reward brain regions, such as the striatum [6]. To compensate for this dopaminergic overstimulation, the striatum exhibits less D2/D3 receptors [7], [8], [9]. This reduction in D2/D3 receptor density seems to trigger further overeating, since a lower calorie intake would, at this point, result in an insufficient hedonic response [10]. In line with these findings, we recently identified altered gray matter density (GMD) in the ventral striatum (i.e., nucleus accumbens) and the posterior medial orbitofrontal cortex in relation to an increasing body-mass index (BMI) and leptin serum concentration [11]. Both regions are known as crucial nodes within the brain's reward circuitry [12]. Leptin is a circulating adipocyte-derived hormone that correlates strongly with the amount of body fat [13], [14]. Women showed additional GMD changes in the dorsal striatum (i.e., putamen) and the dorsolateral prefrontal cortex (DLPFC) suggesting distinct gender-related influences of obesity on brain regions involved in habit learning and executive control [11].

Only few studies investigated the influence of obesity on WM structure [15], [16]. Using diffusion-tensor imaging (DTI) and tract-based spatial statistics (TBSS), Stanek and colleagues [17] correlated the fractional anisotropy (FA) with the BMI. They found that an increasing BMI was associated with lower FA in the genu, splenium, and fornix, while a BMI-by-age interaction emerged for FA in the splenium and body of the corpus callosum. These results suggest an association between obesity and diffusion parameters. In a parallel DTI study of obesity-associated alterations in WM structure, we obtained consistent results [18]. Moreover, we further found reduced radial diffusivity, λ_⊥_, in the corpus callosum that was significantly correlated with an increasing BMI and leptin serum concentration in women, whereas significance was not reached in men [18]. Changes in λ_⊥_ were stronger in the anterior sub-regions of the corpus callosum. These findings, together, share some similarities with WM-related findings due to brain aging [5].

In search of a potential interrelationship between obesity-associated alterations in GMD and diffusion parameters, we performed further analyses of data obtained from lean to obese women in both our recent studies [11], [18]. Here, we hypothesized a significant correlational relationship between GMD alterations in frontal and striatal brain regions, and changes of DTI parameters in the corpus callosum in obesity. We only included females, since the female sub-cohort of our recent study showed body weight related GMD alterations in the left putamen and the right DLPFC [11] and correlations between obesity and diffusivity parameters (FA, λ_⊥_) in the corpus callosum [18].

## Materials and Methods

### Data acquisition

Data of 21 young lean to obese female volunteers (age 24.9±3.9 y; BMI 29.1±7.6 kg/m^2^, range 18.5–45.4 kg/m^2^), who had participated in previous studies [11], [18], were analyzed to investigate a body-weight specific interrelationship between GM and WM structure. Briefly, exclusion criteria were depression (Beck's Depression Inventory (BDI), cut-off value 18), a history of neuropsychiatric diseases, smoking, diabetes mellitus, hypertension, conditions, which are contraindications to magnetic resonance imaging (MRI), and abnormalities in the T1-weighted MRI scan upon visual inspection. The study was carried out in accordance with the Declaration of Helsinki and had been approved by the Ethics Committee of the University of Leipzig. Subjects gave their written informed consent prior to participation.

Scanning was performed on a 3-T TIM Trio (Siemens, Erlangen, Germany) using a 12-element head matrix coil. Three-dimensional (3D) T1-weighted datasets were acquired with the magnetization-prepared rapid gradient echo (MP-RAGE) sequence with selective water excitation and linear phase encoding [19]. We used a sagittal slice orientation and the following imaging parameters: inversion time (TI) 650 ms; repetition time (TR) 1300 ms; echo time (TE) 3.5 ms; excitation pulse flip angle 10°; bandwidth 190 Hz/pixel; image matrix 256×240; field of view (FOV) 256 mm×240 mm; nominal spatial resolution 1 mm×1 mm×1 mm; 2 averages.

Diffusion-weighted images were acquired from 72 axial slices (thickness 1.72 mm; no gap) with a twice-refocused spin-echo echo-planar-imaging sequence [20], TE 100 ms, TR 12 s, bandwidth 1345 Hz/pixel, image matrix 128×128, FOV 220 mm×220 mm, 60 diffusion-encoding gradient directions, *b*-value 1000 s/mm^2^. Seven images without diffusion weighting were additionally acquired and used for the apparent diffusion coefficient (ADC) computation and as anatomical reference for offline motion correction; one at the beginning of the scanning sequence and one after each block of 10 diffusion-weighted images. Random noise was reduced by averaging three acquisition cycles, resulting in a total acquisition time of about 45 min.

Before the MRI session, BMI was computed using body size and body weight obtained with digital scales. In order to investigate a correlation between BMI and various serum markers, blood from a peripheral venous puncture in the elbow flexure was withdrawn into a serum vacutainer and centrifuged at 4°C for 10 minutes at a relative centrifugal force of 3500 g to separate the serum, which was stored at −80°C. Finally, serum concentrations were determined for insulin, proinsulin, glucose, leptin, soluble leptin receptor (sOB-R), triglycerides, albumin, protein, brain-derived neurotrophic factor (BDNF), C-peptide, cholesterol, low-density lipoprotein (LDL), high-density lipoprotein (HDL), thyroid-stimulating hormone (TSH), cortisol, creatinine, chloride (Cl), potassium (K), sodium (Na), and osmotic concentration (Table S1). Serum leptin concentrations were determined by enzyme-linked immunosorbent assays (Mediagnost, Reutlingen, Germany). In addition, using standardized questionnaires, we also acquired behavioral parameters such as cognitive restraint, compulsive behavior, disinhibition, and hunger. Finally, we assessed parameters for graduation and living conditions (Table S2).

### Data analysis

A correlation analysis was performed to investigate the relationship between BMI and all 20 serum markers listed in Table S1 using the Pearson correlation. Significant correlations were obtained with *p*<0.05 (2-tailed) using Bonferroni correction for multiple comparisons resulting in a significance level of 0.0025. We also tested for significant correlation between BMI and behavioral parameters listed in Table S2. Using four behavioral parameters, correction for multiple comparisons resulted in a significance level of 0.0125.

Although no subject showed a total BDI score above 15, we performed a correlation analysis between all BDI scores (cognitive-affective, somatic, and total BDI) and obesity parameters (BMI, leptin concentration) to investigate a potential relationship between body-weight and mood.

T1-weighted images were processed using SPM8 (Wellcome Trust Centre for Neuroimaging, UCL, London, UK) and Matlab 7 (Mathworks, Sherborn, MA, USA). We used the default unified segmentation approach for segmentation, bias-correction, and normalization [21]. Subsequently, a fast diffeomorphic image registration algorithm called Diffeomorphic Anatomical RegisTration using Exponentiated Lie algebra (DARTEL) [22] was applied in order to generate a group-specific DARTEL template of gray matter (GM) tissue. All individual GM segments were warped to the DARTEL template and modulated by the Jacobian determinants of deformations introduced by normalization to account for local compression and expansion during transformation. Finally, a Gaussian filter of 8 mm full width at half maximum (FWHM) was applied. In agreement with general practice, we used the term *gray matter density* (GMD) to describe the modulated and normalized GM probability values.

Voxel-wise statistical analyses were performed twice using the general linear model, either including serum leptin concentrations or BMI as covariates of interest. Both models also included covariates of no interest for age and total GM and WM volumes to account for the confounding effects of age and brain size. Parameters were estimated for all voxels with a minimum GMD of 20%. To replicate voxel-based morphometry (VBM) findings of our recent study [11], we assessed the relationship between GMD and obesity makers (i.e., serum leptin concentration and BMI) in the same way. To this end, we computed a negative correlation between GMD and serum leptin concentrations, and a positive correlation between GMD and BMI using a voxel threshold of *p*<0.001 and a cluster threshold of *p*<0.05, family-wise-error (FWE) corrected. Based on the resulting statistical parametric maps, regions of interest (ROIs) were defined for significant GMD clusters. GMD values from ROIs were extracted and correlated with diffusivity parameters obtained by DTI.

Diffusion-weighted data were processed using the FMRIB Software Library (FSL) [23]. Motion correction was performed with rigid-body transformation [24] using the reference images acquired without diffusion weighting during DTI. This processing step was combined with a global registration to the T1-weighted images. Then diffusion-weighted images were skull-stripped using the individual T1-weighted images and finally co-registered to the standard Montreal Neurological Institute (MNI) template. The gradient direction for each volume was corrected using the rotation parameters. The registered images were interpolated to the new reference frame with an isotropic voxel dimension of 1 mm, and the three corresponding acquisitions and gradient directions were averaged. Finally, for each voxel, a diffusion tensor was fitted to the data. All diffusion parameters (i.e., ADC, FA, axial diffusivity (λ_||_), and λ_⊥_) were computed from the eigenvalues of the diffusion tensor.

Voxel-wise diffusion parameters (ADC, FA, λ_||,_ and λ_⊥_) were statistically analyzed using TBSS [25] as implemented in FSL [23]. For this purpose, a mean FA image was created and thinned to create a mean FA skeleton, which represents the centers of all tracts across subjects. Subjects' diffusion parameters were projected onto this skeleton and analyzed with voxel-wise cross-subject statistical randomization tests [26] to correlate diffusion parameters with GMD values of ROIs as identified with the VBM analyses (see above). This analysis was performed on the whole skeleton. Significant correlations were detected using threshold-free cluster enhancement (TFCE) and correction for multiple comparisons at the *p*<0.05 (corrected) level [27].

Note, that correlation between GMD and white matter diffusivity parameters does not necessarily provide information about direct fiber connections. Therefore, to further elucidate the structural interconnectedness of those callosal regions that showed a correlation with the leptin-related GMD in the DLPFC, we used the measure of connection probability *Px* obtained by the original diffusion weighted data using probabilistic fiber tracking. In addition to consider diffusivity parameters described above, fiber tracking was conducted with the FDT toolbox of FSL [23]. Two fibers were estimated for each voxel unless prevented by automatic relevance detection [28] in order to model crossing fiber architectures. Thereafter, for each subject, probabilistic fiber tractography was computed using the software module PROBTRACKX [28] with using a pre-defined ROI as seed mask covering the anterior half of the corpus callosum. The seed mask was chosen to cover the voxels showing a negative correlation between λ_⊥_ and GMD in the right DLPFC. As target space, the entire brain volume was selected. The *connection probability* was computed by the number of tracts that reach a target voxel from all seed voxels. We used standard parameters with 5000 sample tracts per seed voxel, a curvature threshold of 0.2, a step length of 0.5, and a maximum number of steps of 2000.

Note that all fiber-tracking analyses were conducted in the individual native space. To generate the seed mask for each subject, the ROI in the anterior corpus callosum was transformed to the individual space using linear and non-linear registration between the mean and the individual FA images. The transformed seed masks were further restricted to voxels showing a minimum FA of 0.6. To account for difference in size of the individual seed masks, we divided the connection probability *Px* by the number of voxels in the seed masks [29], [30]. To localize differences in connectivity on the group level, we followed the method proposed by Argyelan and colleagues [31] and adapted it to the characteristics of our data. Therefore, connection probability maps were transformed to the MNI space and smoothed using a Gaussian filtering of 6-mm FWHM to enable a voxel-wise analysis of connectivity across subjects. Correlations between connection probability and the individual serum leptin concentration were computed with randomization tests [26] using a design matrix containing the subjects' leptin levels as a covariate. Significant correlations were detected with *p*<0.01. Note that we did not perform a distance correction because we did not compare the connection probability across regions.

The same technique of probabilistic fiber tracking was also applied to more posteriorly located seeds in the corpus callosum seeking for connecting white matter fibers not only between the corpus callosum and the DLPFC, but also to the putamen.

## Results

We found a significant correlation between BMI and serum leptin concentration (*r* = 0.71) suggesting a higher amount of body fat with higher BMI. Contrarily, we did not find any significant correlation between BMI and any other serum marker (see Table S1). Investigating the relationship between BMI and behavioral parameters (see Table S2) showed a significant correlation between BMI and disinhibition (*r* = 0.59) reflecting a higher degree of disinhibited eating behavior associated with an increased BMI. However, no significant correlation between BMI and any other parameters were found. We also did not find a significant correlation between BDI scores (cognitive-affective, somatic, and total BDI) and obesity parameters (BMI and leptin concentration) suggesting that in our female cohort, overweightness or obesity was not directly associated with depression. All *p*-values were above 0.18.

**Figure 1:**
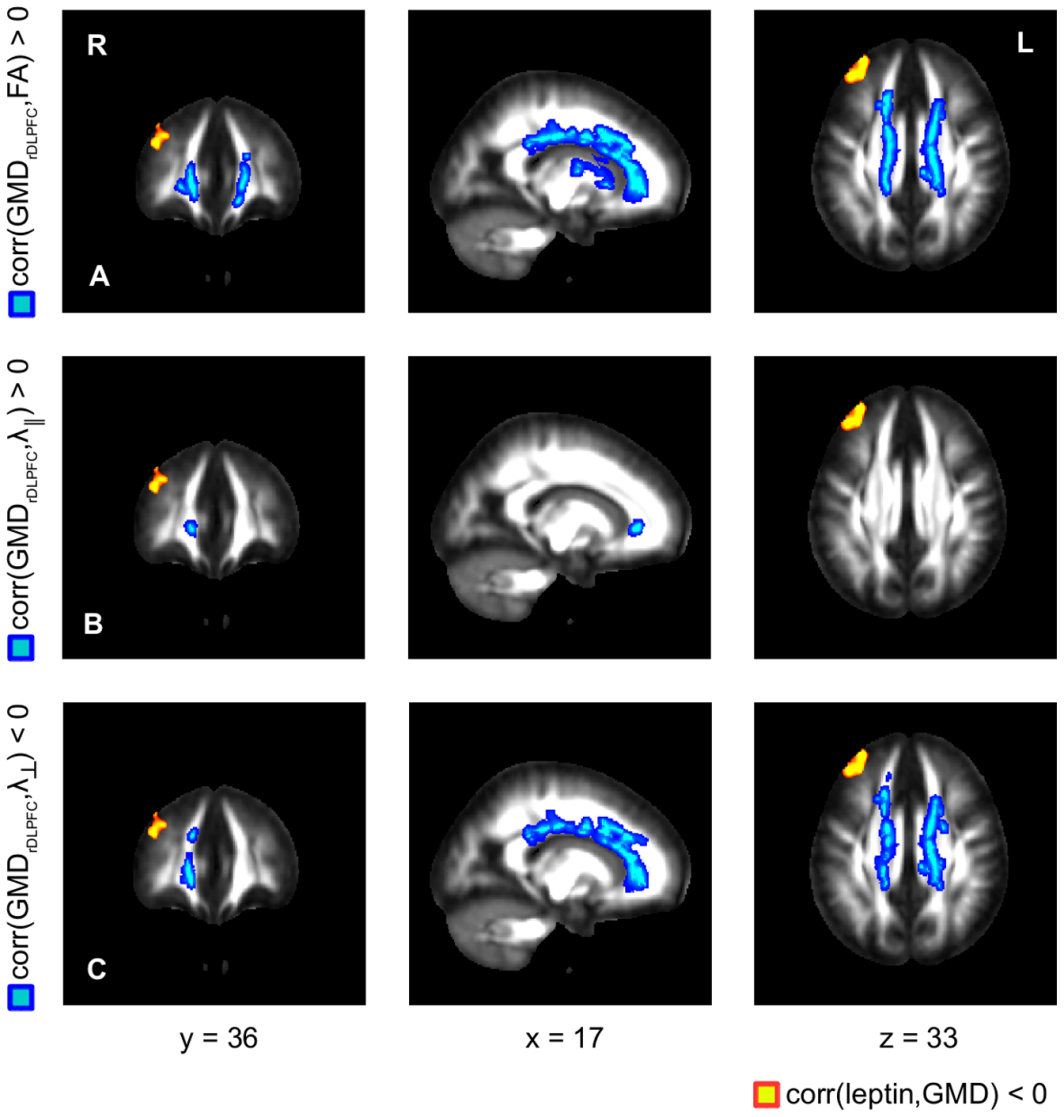
Correlation between GMD_rDLPFC_ and diffusion parameters. Voxel-based morphometry yielded a negative correlation between GMD and the serum leptin level in the right DLPFC (color-coded in red/yellow, *p*<0.001). Using randomization tests, a significant positive correlation was found between GMD_rDLPFC_ and FA in the corpus callosum (**A**; color-coded in blue, *p*<0.05, corrected). Inspection of the diffusion tensor elements (**B, C**) indicated that the decreased FA observed for a decreased GMD_rDLPFC_ predominantly resulted from an increased λ_⊥_ (**C**; negative correlation between λ_⊥_ and GMD_rDLPFC_, *p*<0.05, corrected), whereas indications of decreased λ_||_ were limited to a small region in the genu of the corpus callosum (**B**; positive correlation between λ_||_ and GMD_rDLPFC_, *p*<0.05, corrected). No significant correlation was observed between GMD_rDLPFC_ changes and ADC.

The VBM analysis for the 21 lean to obese women revealed a significant negative correlation between serum leptin levels and GMD in the right DLPFC (GMD_rDLPFC_) using a voxel threshold of *p*<0.001 and a cluster threshold of *p*<0.05, FWE-corrected (Figure 1, color-coded in red/yellow). The Pearson correlation coefficients were *r* = −0.48 between serum leptin levels and GMD_rDLPFC_ and *r* = −0.58 between BMI and GMD_rDLPFC_.

Next, we correlated GMD_rDLPFC_ with all tensor-based diffusion parameters in the entire WM. The randomization tests within the TBSS framework revealed a positive correlation between GMD_rDLPFC_ and FA (*p*<0.05, TFCE-corrected, Figure 1A, color-coded in blue) and a negative correlation between GMD_rDLPFC_ and λ_⊥_ (Figure 1C) along the entire corpus callosum, while GMD_rDLPFC_ was positively correlated with λ_||_ only in a small region located in the genu (Figure 1B). We did not observe any significant correlation between GMD_rDLPFC_ values and ADC across the entire brain.

**Figure 2:**
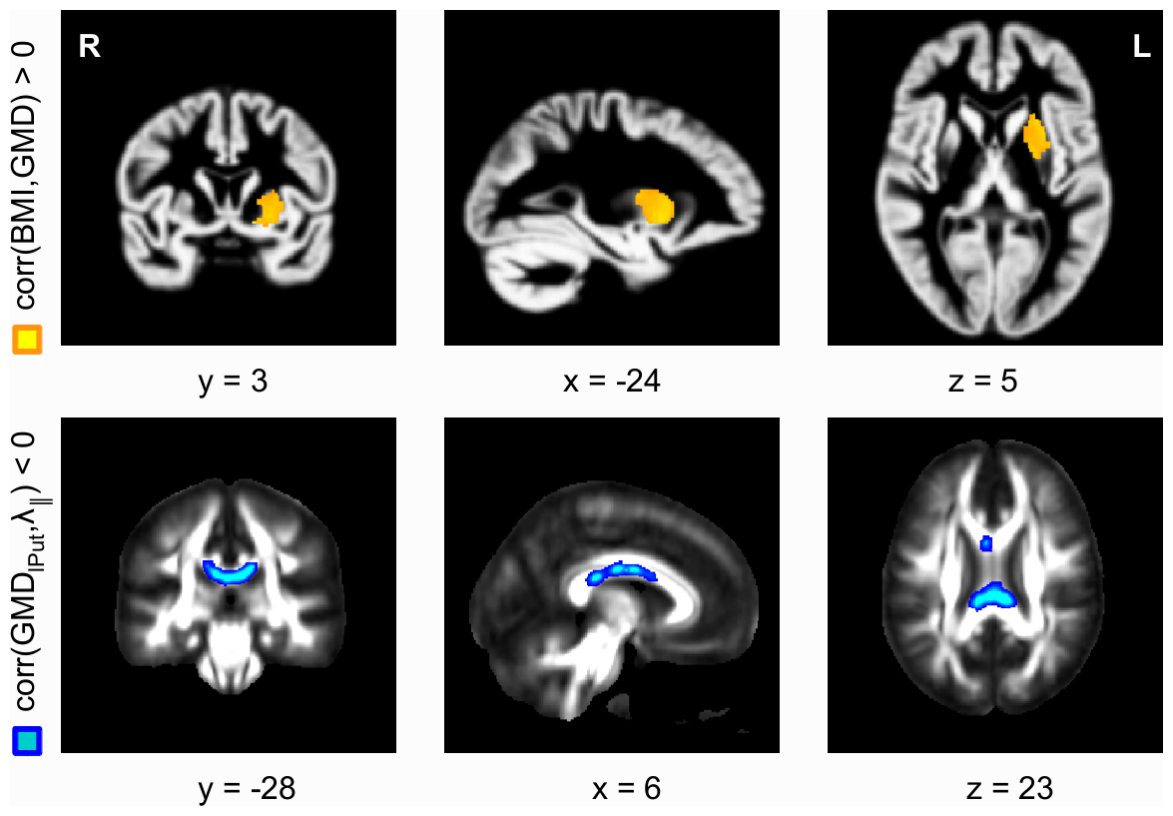
Correlation between GMD_lPut_ and λ_||;_. A positive correlation was observed between BMI and GMD in the left putamen (**A**; color-coded in yellow, *p*<0.05, FWE-corrected). λ_||;_ was negatively correlated with GMD_lPut_, predominantly in posterior and inferior areas of the body of corpus callosum (**B**; color-coded in blue, *p*<0.05, TFCE-corrected). Correlations between GMD_lPut_ and the other diffusion parameters, FA, λ_⊥_, and ADC, did not reach significance.

The VBM analysis also showed a significant positive correlation between BMI and GMD in the left putamen (GMD_lPut_) using a voxel threshold of *p*<0.001, and a cluster threshold of *p*<0.05, FWE-corrected (Figure 2A, color-coded in yellow). The Pearson correlation coefficients were *r* = 0.67 between BMI and GMD_lPut_ and *r* = 0.68 between serum leptin levels and GMD_lPut_. We also obtained a significant correlation between BMI and GMD in the right putamen (GMD_rPut_) with *p*<0.001 on the voxel level, however, the cluster was not significant with FWE-correction. The Pearson correlation coefficients were *r* = 0.36 between BMI and GMD_rPut_ and *r* = 0.46 between serum leptin levels and GMD_rPut_. As for the GMD_rDLPFC_, we used randomization tests to correlate tensor-based diffusion parameters with GMD_lPut_ across the whole WM skeleton. We found a significant negative correlation between GMD_lPut_ and λ_||;_ predominantly in posterior and inferior areas of the body of the corpus callosum, *p*<0.05, TFCE-corrected (Figure 2B, color-coded in blue). We found no significant correlation between GMD_lPut_ and the other diffusion parameters ADC, FA, and λ_⊥;_. Recently, we had observed further positive correlations of BMI and GMD in the ventral striatum (i.e., nucleus accumbens) and in the posterior medial orbitofrontal cortex [11]. In these regions, however, significance was not reached in the present subgroup.

**Figure 3:**
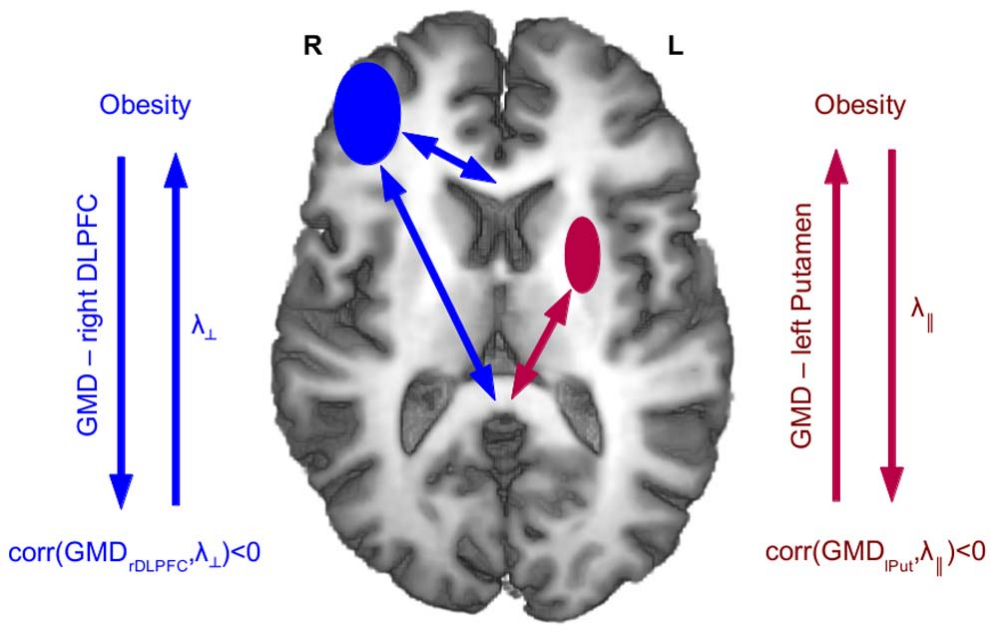
Schematic summary of correlation between diffusion parameters in the corpus callosum and GMD values in the left putamen and right DLPFC (see also Figures 1 and 2). GMD_rDLPFC_ was negatively correlated with λ_⊥;_ in genu and splenium of the corpus callosum (blue arrows), while the GMD_lPut_ was negatively correlated with λ_||;_ in the body and splenium (red arrows). Thus, an obesity-related reduction of GMD in the right DLPFC was associated with an increase of λ_⊥;_ in the entire corpus callosum, while an obesity-related increase of GMD in the left putamen was associated with a decrease of λ_||;_ in the body and splenium of the corpus callosum.

Taken together, in the current female cohort, we found different correlations between diffusion parameters and GMD in the DLPFC and in the dorsal striatum: Gray-matter density in the right DLPFC correlated negatively with λ_⊥;_ along the corpus callosum (Figure 3, blue arrows) indicating an obesity-related GMD decrease in the right DLPFC parallel to an increased λ_⊥;_. In the left putamen, however, GMD showed a negative correlation with λ_||;_ predominantly in posterior parts and inferior areas of the body of the corpus callosum (Figure 3, red arrows) indicating an obesity-related GMD increase in the left putamen in parallel to a decreased λ_||;_ within the body and splenium.

**Figure 4:**
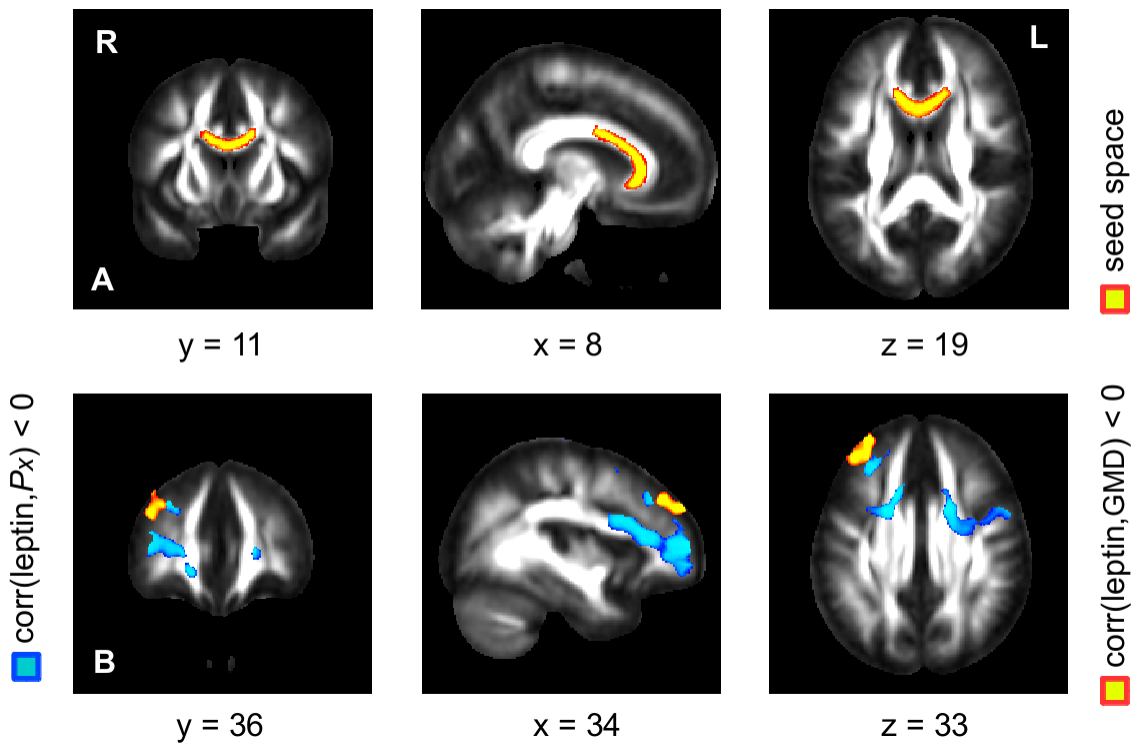
Correlation between serum leptin concentration and connection probability *Px.* Probabilistic fiber tracking was performed using a seed mask in the anterior corpus callosum (**A**; color-coded in yellow). Higher serum leptin concentrations were associated with smaller connection probabilities between the anterior corpus callosum and prefrontal WM regions (**B**; color-coded in blue). Significant correlation was detected using randomization tests with a significance level of *p*<0.01. The detected prefrontal WM regions are located in the vicinity of the observed GM regions showing a negative correlation between leptin level and GMD (**B**; yellow color; see also Figure 1).

Using probabilistic fiber tracking, we obtained a relationship between serum leptin level and connectivity between corpus callosum and the right DLPFC using the measure of connection probability *Px*. Non-parametric statistical analysis showed a significant negative correlation between the serum leptin concentration and connection probability between the anterior corpus callosum (Figure 4, top row, yellow color) and various prefrontal WM regions (Figure 4, bottom row, blue color). These prefrontal WM regions were detected in the vicinity of the observed GM regions showing a negative correlation between GMD and leptin concentration. Thus, subjects with higher leptin level showed both a diminished GMD in the right DLPFC as well as a diminished connection probability between the anterior corpus callosum and prefrontal WM regions.

Note that we did not obtain a significant correlation between leptin level and connection probability between the anterior corpus callosum and left putamen. Applying the same technique of probabilistic fiber tracking to more posteriorly located seeds in the corpus callosum, we did not obtain any significant correlation between leptin concentration and connection probability.

## Discussion

In the present study, we investigated the relationship between obesity-related alterations of GMD and diffusion parameters in WM in a lean to obese female cohort. First, we revisited previous analyses [11], [18] and replicated the negative correlation of leptin serum concentration and GMD changes in the right DLPFC (see Figure 1), as well as the positive correlation of BMI and GMD changes in the left putamen (see Figure 2) for the current female sub-cohort. As discussed recently [11], these findings suggest that obesity is related to GM alterations of brain regions involved in executive control (DLPFC) and habit learning (putamen) [32].

Regarding the relationship between obesity-related GM and WM changes, GMD in the right DLPFC was negatively correlated with λ_⊥;_ (Figure 1C), suggesting that the obesity-related GMD_rDLPFC_ reduction is accompanied by structural differences in the entire corpus callosum. Since probabilistic fiber tracking confirmed the relationship between serum leptin level and connectivity between corpus callosum and the right DLPFC, we assume that the large spatial spread of the DLPFC-correlation may be due to the strong whole-brain interconnectedness of this executive control region, not only within but also across hemispheres [33]. Besides the DLPFC we also tested for a correlation between GMD in the left putamen (GMD_lPut_) and λ_⊥;_, but did not find a significant correlation suggesting different obesity-related mechanisms leading to correlated GM and WM changes. However, that might be a false negative finding due to the small sample size and, hence, low statistical power.

Previously, λ_⊥;_ has been proposed to be a putative myelin marker based on studies in a shiverer mouse model [34]. However, others have stressed that myelination is not the only parameter with an effect on radial diffusivity [35] because multiple factors can produce variations of λ_⊥;_ or FA, including alterations in the fiber density, fiber diameter, or membrane permeability [36]. The assumption that the callosal WM changes observed here might include myelin-related processes thus remains speculative, and the use of other modalities, for example relaxographic imaging [37], [38] or quantitative magnetization-transfer imaging [39], [40], is advocated for future studies of obesity as potentially more specific measures of myelination. A (positive) correlation of λ_||;_ and GMD_rDLPFC_ was obtained only in the genu of the corpus callosum (Figure 1B), which might point to enhanced structural changes in this sub-region. Finally, we note that both an increased λ_⊥;_ and a decreased λ_||;_ contribute to a reduction in FA, consistent with the experimental observation (Figure 1A). We also found a significant negative correlation between GMD in the left putamen and λ_||;_, predominantly in posterior regions and inferior areas of the body of the corpus callosum, suggesting that the obesity-associated increase in GMD in the dorsal striatum parallels structural WM changes within these callosal fiber bundles (Figure 2B).

To assess whether the GMD-related alterations of WM fiber bundles within the corpus callosum connect to the leptin-related, altered GM structures within putamen and right DLPFC, we computed connection probabilities obtained by seed-based probabilistic fiber tracking. Placing the seed in the anterior part of the corpus callous (Figure 4, top row), we identified fiber bundles reaching towards the substructure within the right DLPFC that we also found to be altered in relation to the serum leptin levels (Figure 4, bottom row, blue color). These findings suggest that females with higher levels of serum leptin (indicating elevated body fat) did not only present reduced GMD in the right DLPFC but also lower probabilities of structural interconnectedness with the anterior corpus callosum. Note that we did not obtain a significant correlation between serum leptin concentration and the connection probability between corpus callosum and putamen. Previous parcellation studies in healthy individuals showed that the frontal lobe fibers predominantly occupy the genu and the more anteriorly located part of the body [41], [42], while fiber locations for the subcortical nuclei are highly variable but mostly occupy the inferior and posterior areas of the body of corpus callosum [42]. The lower local specificity of the latter subcortical-callosal connections may explain why we did not find probabilistic tracts from further posteriorly located seeds in corpus callosum targeting the putamen.

The local expansion of diffusion parameters correlating with GMD changes in the right DLPFC as well as their relationship to λ_⊥;_ largely replicates previous observations on obesity-related changes in the corpus callosum's apparent water diffusivity [17], [18]. In a larger cohort that also included the current study's sub-cohort, we had shown that λ_⊥;_ significantly increased with increasing BMI (and leptin levels) in women. These changes were comparable to those reported for the aging brain [5], but their specific evolution remained speculative [18]. Here, we show a correlation between obesity-related GMD changes in the right DLPFC in women and diffusion parameters suggesting a mutual relationship between obesity-related alterations of a specific cortical region, known for its involvement in behavioral control, and their topographically associated fiber bundles within the corpus callosum (Figures 1–3). The observed WM changes share similarities with aging-related changes in diffusion parameters in women, also predominantly affecting λ_⊥;_. However, we have to stress that changes in the radial diffusivity are too unspecific for reliably inferring mechanisms of microstructural changes in the fiber architecture. Nevertheless, aging seems to affect water diffusion not only in the corpus callosum but more generally in the entire brain WM skeleton [4]. This might suggest that (in women) obesity may accelerate “aging” of WM structures, however, not across the entire brain but rather of fiber bundles within corpus callosum topographically related to brain regions involved in controlling eating behavior. However, the question remains if our observed relationship between GMD in the right DLPFC and λ_⊥;_ in the corpus callosum is really specific to these regions or just detected due to an increased sensitivity of the method because of an enormous amount of parallel axons in the corpus callosum. In the literature, there is controversial evidence whether obesity affects the entire brain or specific regions. In previous studies, obese individuals showed smaller total brain volume [3] and an increase in WM hyper-intensities, but also locally specific structural alterations, such as a decrease in hippocampal volume [2]. Structural MRI studies with voxel- and tensor-based methods allowed for a more detailed assignment of obesity-related structural brain changes. Walther et al. [43] unveiled a negative correlation between GMD and BMI in the orbitofrontal cortex, which is involved in the representation of flavor and rewarding properties of food [44]. Pannacciulli et al. confirmed this negative correlation between GMD and BMI in brain areas involved in reward processing, but showed further correlations in brain regions underpinning the regulation of taste, and behavioral control [45], [46]. The direction of correlation between BMI and GMD in central reward regions as reported in these studies does not completely match the findings obtained from our cohort. In particular, we also found a negative correlation for the right DLPFC, but a positive instead of a negative correlation for the putamen, furthermore with distinct differences between females and males [11]. The differences between our and others' findings may thus result from the fact that we here exclusively investigated females, whereas other studies (see previous paragraph) did not specifically control for the factor ‘gender’. Finally, we cannot rule out that methodological inconsistencies among studies might contribute to different observations as a recent evaluation demonstrated a significant impact from both the image acquisition as well as the specific segmentation routine on VBM results [47]. Further work should also include parameters of systematic inflammation to assess possible links between obesity-related alterations in brain structure and inflammation processes [48].

## Conclusion

In the context of obesity, we found a relationship between gray and white matter brain regions associated with a higher amount of body fat as measured by the serum leptin concentration. Specifically, we observed correlations between WM diffusivity parameters and GMD in the right DLPFC and left putamen. However, both regions were differently related to body fat content. Our findings suggest GMD differences within the right DLPFC that were negatively correlated with radial diffusivity of interconnected fiber bundles in the anterior corpus callosum (Fig. 3, blue arrows). Further evidence was found using probabilistic fiber tracking showing a leptin-dependent connection probability between corpus callosum and prefrontal WM regions (Fig. 4). The other GM brain structure that we found altered in relation to an elevated body fat content was the left putamen, however, exhibiting a different pattern of structural alterations. The putamen showed a leptin-related increase in GMD, which was negatively correlated with a reduced axial diffusivity in the splenium of the corpus callosum (Fig. 3, red arrows). Together, these findings suggest that an increasing body fat content parallels complex plastic adaptations of GM together with WM structure.

## Supporting Information

Table S1 **Cohort description including age, body weight and height, body-mass index (BMI), waist-to-hip ratio (WHR), and further blood serum concentrations of insulin, proinsulin, glucose, leptin, soluble leptin receptor (sOB-R), triglycerides, albumin, protein, brain-derived neurotrophic factor (BDNF), C-peptide, cholesterol, low-density lipoprotein (LDL), high-density lipoprotein (HDL), thyroid-stimulating hormone (TSH), cortisol, creatinine, chloride (Cl), potassium (K), sodium (Na), and osmotic concentration.**
(XLS)

Table S2 **Cohort description including behavioral parameters as cognitive restraint, compulsive behavior, disinhibition, hunger, and parameters for graduation and living conditions.**
(XLS)
